# How negative parenting relates to adolescents’ perceptions of older adults: the roles of empathy and intergenerational contact

**DOI:** 10.3389/fpsyg.2026.1867088

**Published:** 2026-09-28

**Authors:** Jing Lin, Chao Pan, Dongdong Gao

**Affiliations:** 1 Law School, Ningbo University, Ningbo, China; 2 School of Public Affairs, Zhejiang University, Hangzhou, China; 3 School of Psychology, Henan University, Kaifeng, China

**Keywords:** Adolescent, empathy, intergenerational contact, negative parenting, stereotype content model

## Abstract

**Introduction:**

Grounded in social learning theory, this study examines how negative parenting practices are associated with adolescents’ perceptions of older adults and investigates the roles of empathy and intergenerational contact in this process.

**Methods:**

Data were collected from 2,011 Chinese adolescents using a three-wave longitudinal design. Structural equation modeling was used to test the hypothesized relationships.

**Results:**

Negative parenting was significantly associated with less favorable evaluations of older adults on both the warmth and competence dimensions. Empathy mediated these relationships. Intergenerational contact further moderated the association between empathy and adolescents’ perceptions of older adults. Specifically, the indirect effects of negative parenting through empathy varied according to adolescents’ levels of intergenerational contact.

**Discussion:**

These findings highlight family socialization as an important developmental context for adolescents’ perceptions of older adults and identify empathy as a key socio-emotional mechanism linking parenting experiences to intergenerational cognition.

## 1 Introduction

The accelerating global population aging has brought intergenerational relations and age-related prejudice to the forefront of public and academic attention. In East Asian societies deeply influenced by Confucian family culture, particularly China, the cultural norms of respecting elders and practicing filial piety have long been embedded in the social ethical system (Park and Chesla, 2007). This has led to widespread societal expectations that adolescents should demonstrate respect and positive attitudes toward older adults. However, recent surveys have indicated a rising trend of negative perceptions among adolescents toward older adults, with stereotypes such as “low competence,” “rigid thinking,” and “social burden” becoming increasingly prevalent (Felc and Felc, 2025). This discrepancy between traditional cultural ideals and actual cognitive biases not only deviates from traditional ethical expectations but also hinders the construction of an active aging society, underscoring the need to clarify the processes through which adolescents develop negative views of older adults. In the Chinese context, however, filial piety is not merely a cultural norm but is primarily transmitted through everyday family interactions. Consequently, adolescents’ perceptions of older adults may reflect not only broader cultural expectations but also the ways in which these values are modeled and reinforced within the family.

Age stereotypes refer to simplified and stable beliefs about older adults, often manifested as biased or rigid evaluations (Cuddy et al., 2005). According to the stereotype content model, these stereotypes comprise two dimensions: warmth and competence (Luna Cortes, 2024). The warmth dimension encompasses perceptions of older adults as cold, withdrawn, irritable, or difficult to communicate with, while the competence dimension involves views of older adults as low in ability, highly dependent, and poor in adaptability. Such stereotypes are particularly pronounced among adolescents (Bellingtier et al., 2022). Existing scholarship on age stereotypes has predominantly focused on structural factors (e.g., population aging, labor market competition), media representations of older adults, and direct intergenerational contact experiences (Burn et al., 2022; Wangler and Jansky, 2023). While these factors undoubtedly contribute to age-related prejudice, they largely overlook the critical role of the family—the primary socialization context for adolescents that shapes children’s fundamental beliefs about interpersonal relationships, emotional expression, and social evaluation (Doan et al., 2022; Hu et al., 2024). Surprisingly few studies have examined how early family experiences, particularly parenting practices, influence the development of intergenerational cognition, despite parenting practices providing the foundational templates through which children learn to interact with and evaluate others (Pan, 2025).

The present study addresses these gaps by examining the relationships among negative parenting practices, empathy, intergenerational contact, and adolescents’ warmth and competence stereotypes of older adults using a three-wave longitudinal design with a large sample of 2,011 Chinese adolescents. Specifically, this study highlights family experiences as one important developmental context associated with adolescents’ age stereotypes alongside broader socialization influences, examines empathy as one potential socio-emotional mechanism linking parenting practices to adolescents’ perceptions of older adults, and extends previous research by suggesting that the association between empathy and age stereotypes may vary according to adolescents’ levels of intergenerational contact. Drawing primarily on Social Learning Theory, and using the Stereotype Content Model as an operational framework, this study provides a more comprehensive framework for understanding factors associated with the development of adolescents’ age stereotypes in the Chinese cultural context.

## 2 Literature review and hypothesis development

### 2.1 Social learning theory

This study is grounded in social learning theory (SLT), which posits that individuals learn social behaviors, attitudes, and cognitive styles through observation, imitation, and reinforcement (Bandura, 1977). According to SLT, parents serve as the primary social models for children, and their behaviors and interaction styles are internalized by children as templates for their own social interactions. In the context of this study, negative parenting practices—defined as a set of parenting behaviors characterized by emotional neglect, rejection, harsh criticism, inconsistent discipline, and low emotional responsiveness (Lotto et al., 2023)—provide children with a model of interpersonal interaction marked by coldness and emotional invalidation. Over time, children internalize this interaction style and adopt it as their default approach to relating to others, a pattern that generalizes to interactions outside the family, including those with older adults (Pan, 2025).

SLT also provides a framework for understanding the development of empathy—a multidimensional construct encompassing both affective empathy (the ability to experience others’ emotions) and cognitive empathy (the ability to understand others’ perspectives) (Shamay-Tsoory and Hertz, 2022). Empathy is not an innate ability but is learned through observing and experiencing empathic responses from caregivers (Zhou et al., 2024). When parents consistently validate their children’s emotions, respond sensitively to their needs, and model empathic behavior toward others, children learn to recognize and understand emotions in themselves and others. In contrast, negative parenting practices that neglect or invalidate children’s emotions disrupt this learning process, leading to deficits in empathic ability (Lin et al., 2023; Tang et al., 2023). This theoretical perspective forms the basis for our hypotheses linking parenting practices, empathy, and intergenerational attitudes.

### 2.2 Negative parenting practices and adolescents’ stereotypes of older adults

The first hypothesis of this study concerns the direct relationship between negative parenting practices and adolescents’ age stereotypes—simplified, rigid, and often biased beliefs about individuals based solely on their chronological age. According to the widely accepted stereotype content model (SCM), age stereotypes are structured along two fundamental dimensions: warmth (perceptions of kindness, trustworthiness, and friendliness) and competence (perceptions of ability, intelligence, and efficiency) (Cuddy et al., 2009). While older adults are often viewed as relatively high in warmth, they are consistently stereotyped as low in competence across cultures, a pattern particularly pronounced among adolescents (Bellingtier et al., 2022).

From an SLT perspective, negative parenting practices may contribute to broader interpersonal expectations characterized by reduced trust and emotional responsiveness (Chen et al., 2024; Wray-Lake and Flanagan, 2012). These generalized interpersonal expectations may subsequently be reflected in adolescents’ evaluations of older adults, who represent an important social group in adolescents’ social environments. Additionally, negative parenting practices often involve authoritarian control and a lack of respect for children’s autonomy (Bi et al., 2018), which may lead adolescents to associate age with inflexibility and authoritarianism, reinforcing stereotypes of older adults as rigid and incompetent (i.e., low in competence).

Empirical evidence supports this reasoning. Studies have shown that children who experience harsh or neglectful parenting are more likely to exhibit aggressive behavior, have difficulty forming positive peer relationships, and hold negative attitudes toward outgroups (Agalar et al., 2025; Yang et al., 2023). And the broader literature suggests that negative parenting practices foster a general pattern of negative social cognition that is likely to extend to perceptions of older adults (Pan, 2025; Wang et al., 2022). Based on this theoretical and empirical foundation, we propose:

*H1*: Negative parenting practices are negatively associated with adolescents’ evaluations of older adults on both warmth (H1a) and competence (H1b) dimensions.

### 2.3 The mediating role of empathy

While negative parenting practices may directly influence adolescents’ stereotypes of older adults, we argue that this relationship is primarily mediated by deficits in empathy. This mediating pathway is grounded in two well-established empirical findings: (1) negative parenting practices impair the development of empathy, and (2) empathy is negatively associated with prejudice and stereotyping.

First, extensive developmental research has shown that the quality of parent–child interactions is the single most important predictor of children’s empathic development (Mauroy et al., 2025; Morgan and Fowers, 2022). Children who grow up in environments characterized by emotional neglect, rejection, and harsh discipline do not receive the emotional support and validation necessary to develop secure attachment relationships, which are essential for the development of empathy (Chen et al., 2023). For adolescents, existing research has demonstrated that negative parenting styles can affect the development of their empathetic abilities (Goering and Mrug, 2023).

Second, empathy is widely recognized as a key factor in reducing prejudice and stereotyping (Bobba and Crocetti, 2022). Individuals with high levels of empathy are better able to take the perspective of others, recognize their individuality, and understand their experiences. This perspective-taking ability reduces reliance on simplistic stereotypes and promotes more nuanced and positive evaluations of outgroup members (Timmer-Murillo et al., 2026). In contrast, individuals with low empathy are more likely to rely on superficial cues and categorical thinking when evaluating others (Rademacher et al., 2026), increasing their susceptibility to stereotyping vulnerable groups such as older adults.

Taken together, these findings suggest that negative parenting practices lead to deficits in empathy, which in turn lead to more negative stereotypes of older adults. This mediating pathway provides a mechanistic explanation for how early family experiences shape intergenerational attitudes. Based on this reasoning, we propose:

*H2*: Empathy mediates the relationship between negative parenting practices and adolescents’ stereotypes of older adults, such that negative parenting predicts lower empathy, which in turn predicts lower evaluations of older adults on both the warmth and competence dimensions.

### 2.4 The moderating role of intergenerational contact

While empathy is an important predictor of positive intergroup attitudes, its effectiveness may depend on the presence of meaningful intergenerational contact—defined as direct face-to-face interactions between individuals from different age groups. Extensive research has shown that positive intergroup contact reduces prejudice by increasing knowledge, reducing anxiety, and promoting empathy (Pettigrew and Tropp, 2008; Tropp et al., 2022). In the context of age relations, intergenerational contact has been shown to improve attitudes toward older adults by humanizing them and challenging negative stereotypes (Cao et al., 2026).

We argue that intergenerational contact moderates the relationship between empathy and stereotypes of older adults, such that empathy has a stronger positive effect on attitudes when intergenerational contact is high. This hypothesis is grounded in the idea that empathy is a latent ability that requires concrete interactional experiences to be activated and directed toward specific groups (Tropp et al., 2022). Even adolescents with high levels of empathy may struggle to apply their empathic abilities to a group they have never interacted with, as they lack the personal experiences necessary to humanize older adults and challenge negative stereotypes (Long et al., 2022).

In contrast, when adolescents have frequent and positive interactions with older adults, they gain firsthand knowledge of their individual characteristics, abilities, and personalities. This personal experience provides a context in which empathy can be activated and applied (Cao et al., 2026). Empathetic adolescents are more likely to notice and respond to the emotions and experiences of older adults during these interactions, leading to more positive and individualized perceptions. Over time, these positive interactions challenge and replace negative stereotypes, resulting in more favorable evaluations of older adults on both the warmth and competence dimensions (Kwong and Yan, 2022). Therefore, we propose:

*H3*: Intergenerational contact moderates the relationship between empathy and adolescents’ stereotypes of older adults, such that the positive effect of empathy on evaluations of older adults’ warmth (H3a) and competence (H3b) is stronger when intergenerational contact is high than when it is low.

In summary, the hypothesized model of this study is presented in Figure 1.

**Figure 1:**
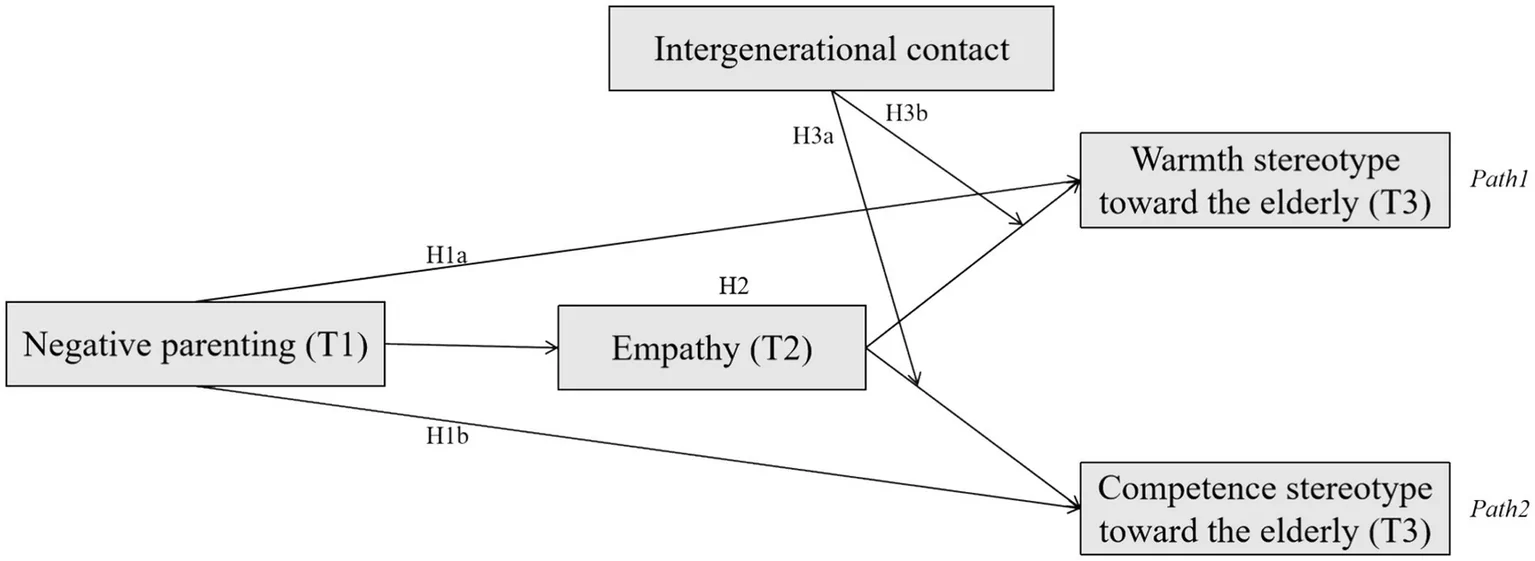
The hypothesis model.

## 3 Method

### 3.1 Participants

Participants were recruited from multiple middle schools in Henan Province, China. After obtaining approval from school administrators and parental consent, random sampling was conducted across the entire student body in each school to collect data. We went into their classes on the spot and distributed paper questionnaires for them to fill out. After filling out, we inputted the results of these questionnaires one by one into SPSS. To reduce common method bias, data were collected at three time points (The interval is 3 months). The student numbers of these adolescents serve as matching identifiers for the data collected at each stage. At Time 1, adolescents completed measures of parental negative parenting practices, intergenerational contact and provided demographic information (2,512 valid questionnaires). At Time 2, they completed measures of empathy (2,336 valid questionnaires). At Time 3, they completed measures of stereotypes of older adults (2,011 valid questionnaires), resulting in an overall effective response rate of 80.01%. Among the 2,011 participants, 55.8% were female, 77.9% were from rural areas, 76.8% were non-only children, and the average age was 14.79 years (SD = 1.49). All participants will fill out an informed consent form before each measurement and are assured that the data will be kept confidential and used only for research purposes.

### 3.2 Measures

#### 3.2.1 Negative parenting practices

The negative parenting subscale from the Chinese version of the Egna Minnen av. Barndoms Uppfostran (EMBU) was used (Li et al., 2012). This subscale consists of 6 items rated on a 4-point Likert scale. The higher the score, the higher the level of negative parenting perceived by adolescents from their parents. The Cronbach’s α coefficient for this scale in the present study was 0.88.

#### 3.2.2 Empathy

The Chinese version of the Interpersonal Reactivity Index (IRI-C), the most widely used measure of empathy, was employed (Zhang, 2025). This scale includes 22 items rated on a 7-point Likert scale. The higher the score, the higher the level of adolescents’ empathy. The Cronbach’s α coefficient for this scale in the present study was 0.91.

#### 3.2.3 Intergenerational contact

Intergenerational contact was measured using a single objective item: “How long have you lived with your grandparents?” (The unit of counting is the year). This approach provides a simple and objective measure of intergenerational contact based on cohabitation duration.

#### 3.2.4 Stereotypes of older adults

Stereotypes were measured using the two-dimensional stereotype content model approach developed in previous research (Cuddy et al., 2009). Participants rated older adults (general) on 12 adjectives, with 6 items measuring the warmth dimension (e.g., kind, sincere, trustworthy, amiable, friendly) and 6 items measuring the competence dimension (e.g., competent, wise, efficient, capable, confident, skilled). All items were rated on a 7-point Likert scale, with higher scores indicating more positive stereotypes on that dimension. The Cronbach’s α coefficients for the warmth and competence dimensions in the present study were 0.81 and 0.88, respectively.

#### 3.2.5 Control variables

Consistent with previous research, several demographic variables were included as controls: gender (1 = male, 2 = female), age (continuous variable), only-child status (0 = no, 1 = yes), residential location (1 = urban, 2 = rural), and family income (continuous variable). Because these variables have been proven to be closely related to an individual’s empathy or stereotypes, etc. (Basyir et al., 2025; He et al., 2025; Zhan et al., 2026).

### 3.3 Data analysis

Data were analyzed using SPSS and Mplus software. First, common method bias was tested, and confirmatory factor analysis was conducted using Mplus. Second, descriptive statistics and correlation matrices were computed. Third, structural equation modeling was employed to test the research hypotheses.

## 4 Results

### 4.1 Common method Bias test

Harman’s single-factor test was used to assess common method bias. The results showed that the first common factor explained only 16.96% of the variance, which is well below the 40% threshold (Podsakoff et al., 2003). Thus, common method bias did not significantly affect the study results, and further analyses could proceed.

### 4.2 Reliability and validity analysis

Reliability analysis indicated that the Cronbach’s α coefficients for negative parenting practices, empathy, warmth stereotypes, and competence stereotypes were 0.88, 0.91, 0.81, and 0.88, respectively. All coefficients exceeded 0.8, demonstrating good internal consistency. Confirmatory factor analysis using Mplus 7.0 revealed that the four-factor model had better fit indices than alternative models (χ^2^/df = 2.66, TLI = 0.93, CFI = 0.91, SRMR = 0.03, RMSEA = 0.05), indicating good discriminant validity among the variables.

### 4.3 Correlation analysis

Pearson correlation coefficients were calculated, and the results are presented in Table 1. Negative parenting practices were significantly negatively correlated with empathy (*r* = −0.38, *p* < 0.001) and with both warmth (*r* = −0.28, *p* < 0.001) and competence (*r* = −0.27, *p* < 0.001) stereotypes of older adults. Empathy was significantly positively correlated with both warmth (*r* = 0.28, *p* < 0.001) and competence (*r* = 0.37, *p* < 0.001) stereotypes.

**Table 1:** Descriptive statistics and correlation matrix.

Variable	M	SD	1	2	3	4	5
1. Negative parenting	1.57	0.55	—				
2. Empathy	3.73	1.35	−0.38***	—			
3. Intergenerational contact	9.98	5.82	−0.11**	0.11**	—		
4. Warmth stereotype	6.03	1.06	−0.28***	0.28***	0.09**	—	
5. Competence stereotype	5.54	1.07	−0.27***	0.37***	0.11***	0.70***	—

**p < 0.01, ***p < 0.001.

### 4.4 Hypothesis testing

Structural equation modeling was conducted using Mplus, and the model demonstrated good fit (χ^2^/df = 2.15, TLI = 0.95, CFI = 0.97, SRMR = 0.03, RMSEA = 0.04). As shown in Table 2 and Figure 2, after controlling for gender, age, only-child status, residential location, and family income, negative parenting was significantly negatively associated with both warmth (*β* = −0.21, SE = 0.08, *p* < 0.001) and competence (*β* = −0.15, SE = 0.08, *p* < 0.001) stereotypes of older adults, supporting Hypothesis 1.

**Table 2:** Results of the structural equation model.

Predictor	Empathy (T2)		Warmth stereotype (T3)		Competence stereotype (T3)	
	*β*	SE	*β*	SE	*β*	SE
Gender (T1)	0.10**	0.10	−0.11**	0.08	−0.08*	0.08
Age (T1)	−0.02	0.02	0.01	0.02	−0.04	0.02
Only-child status (T1)	−0.01	0.14	−0.07	0.11	−0.08	0.11
Residential location (T1)	0.03	0.02	0.01	0.01	0.01	0.01
Family income (T1)	−0.01	0.01	0.02	0.01	0.03	0.01
Negative parenting (T1)	−0.36***	0.01	−0.21***	0.08	−0.15***	0.08
Empathy (T2)	—	—	0.17***	0.03	0.24***	0.03
Intergenerational contact (T1)	—	—	0.08	0.04	0.10*	0.03
Empathy (T2) × Intergenerational contact (T1)	—	—	0.18***	0.02	0.40***	0.01
*R* ^2^	0.15		0.12		0.15	
*F*	127.18***		93.69***		125.47***	

*p < 0.05, **p < 0.01, ***p < 0.001. *β* represents standardized coefficients. The model remained significant with or without control variables, indicating the robustness of the results.

**Figure 2:**
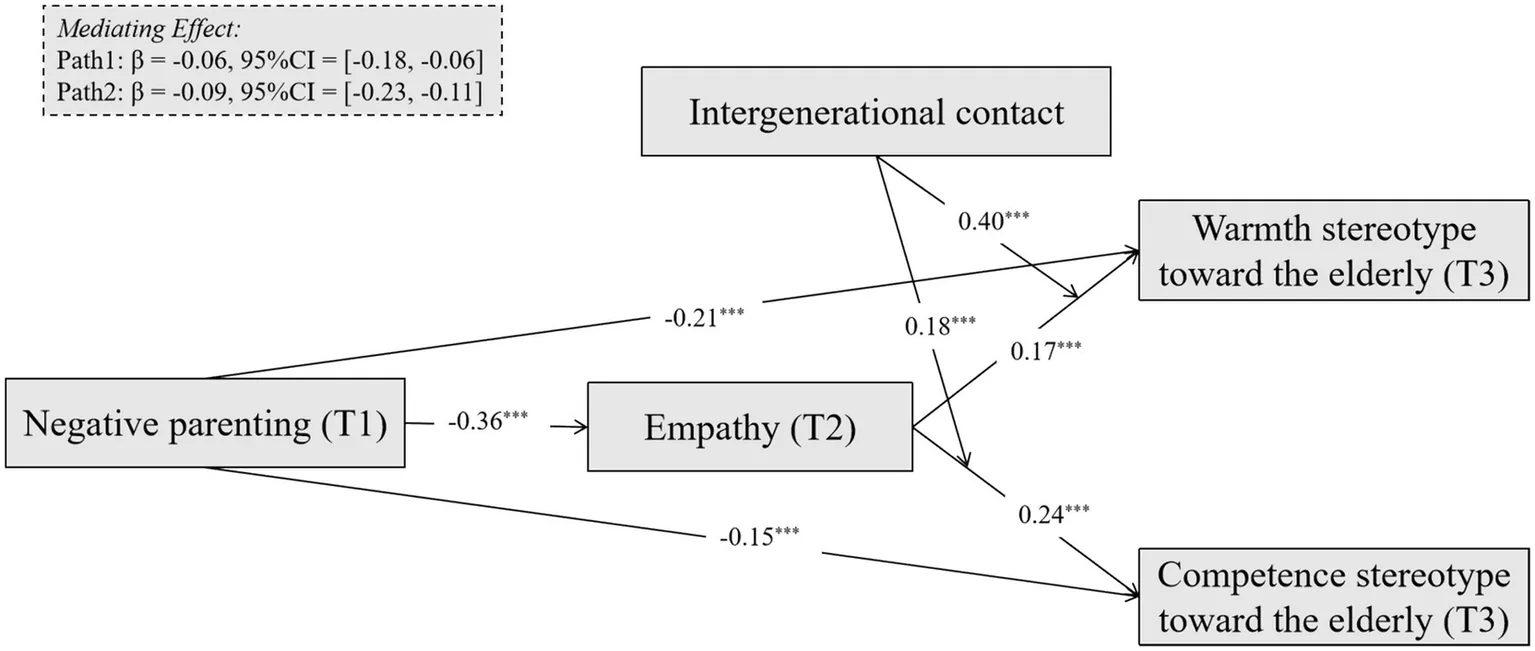
Path coefficient and mediation analysis results.

Second, negative parenting was significantly negatively associated with empathy (*β* = −0.36, SE = 0.00, *p* < 0.001), and empathy was significantly positively associated with both warmth (*β* = 0.17, SE = 0.03, *p* < 0.001) and competence (*β* = 0.24, SE = 0.03, *p* < 0.001) stereotypes. To further test Hypothesis 2, bootstrapping (5,000 times) was used to examine the mediating effect of empathy. The results confirmed that empathy mediated the relationship between negative parenting and both warmth (*β* = −0.06, SE = 0.01, *p* < 0.001, 95% CI = [−0.15, −0.03]) and competence (*β* = −0.09, SE = 0.03, *p* < 0.001, 95% CI = [−0.12, −0.05]) stereotypes of older adults.

Finally, after centralizing the variables, we constructed the interaction terms between empathy and intergenerational contact. The interaction terms were significantly positively associated with both warmth (*β* = 0.18, SE = 0.02, *p* < 0.001) and competence (*β* = 0.40, SE = 0.01, *p* < 0.001) stereotypes, indicating that intergenerational contact moderated the relationship between empathy and stereotypes of older adults. To better visualize the direction of the moderation effect, simple slope analysis was conducted, and the results are presented in Figure 3. For adolescents with low levels of intergenerational contact (M-1SD), empathy significantly positively predicted both warmth (*β* = 0.21, SE = 0.06, *p* < 0.001, 95% CI = [0.10, 0.32]) and competence (*β* = 0.22, SE = 0.06, *p* < 0.001, 95% CI = [0.11, 0.33]) stereotypes. For adolescents with high levels of intergenerational contact (M + 1SD), this positive effect was even stronger (warmth: *β* = 0.33, SE = 0.05, *p* < 0.001, 95% CI = [0.23, 0.42]; competence: *β* = 0.46, SE = 0.05, *p* < 0.001, 95% CI = [0.36, 0.55]). These results supported Hypothesis 3.

**Figure 3:**
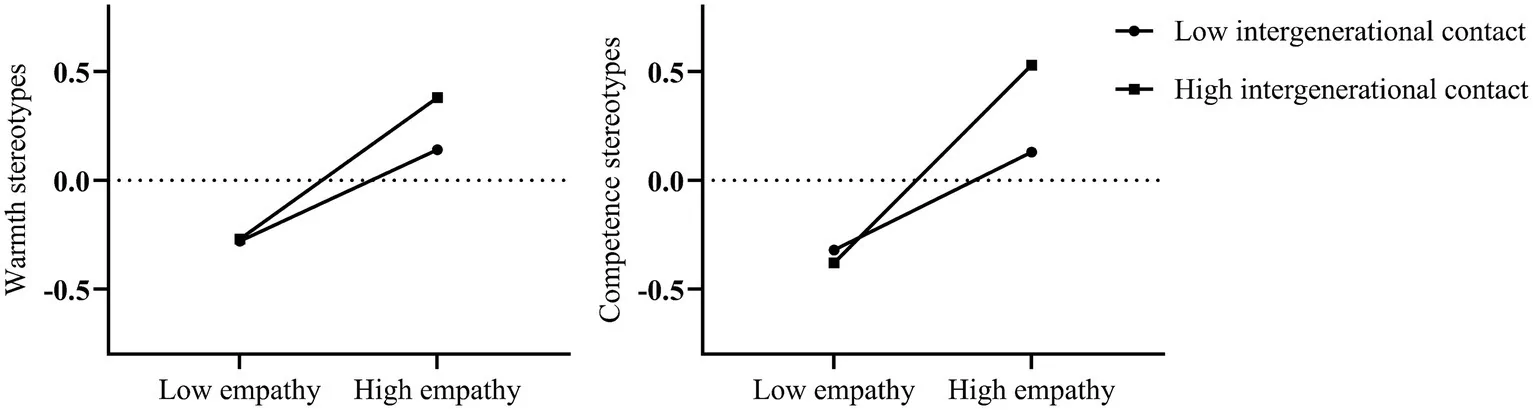
The moderating effect of intergenerational contact.

## 5 Discussion

Grounded primarily in Social Learning Theory, this study examined the relationships among negative parenting practices, adolescents’ empathy, intergenerational contact, and stereotypes of older adults, providing new longitudinal evidence for understanding factors associated with the development of adolescents’ age stereotypes.

First, while previous research has predominantly explained the origins of age stereotypes from perspectives of social structure, media exposure, or direct intergenerational contact (Xu, 2022; Zhang et al., 2022), the present study extends this literature by highlighting family experiences as one important socialization context, alongside schools, peers, and the media, that may be associated with adolescents’ perceptions of older adults. Consistent with Social Learning Theory, parents’ emotional expression and interaction styles provide important developmental experiences through which children learn interpersonal norms and ways of understanding others. Negative parenting practices, characterized by emotional neglect, negative feedback, and low emotional responsiveness, may reduce adolescents’ sensitivity to others’ emotional cues and encourage more detached interpersonal expectations. These interpersonal expectations may, in turn, be associated with lower evaluations of older adults’ warmth and competence. Furthermore, whereas previous research on negative parenting has primarily focused on children’s aggressive behavior, emotion regulation, or peer relationships (Chu and Chen, 2025), relatively little attention has been paid to its potential associations with cross-generational social cognition. The present findings therefore suggest that family experiences may be relevant not only to parent–child relationships but also to adolescents’ evaluations of older adults.

Second, empathy played a pivotal mediating role in this process. This finding is consistent with Social Learning Theory. Within this perspective, Emotionally unsupportive parenting environments may limit adolescents’ emotional resonance and perspective-taking abilities, making them more likely to rely on generalized stereotypes rather than individualized information when evaluating older adults. This interpretation is also consistent with previous research linking parenting styles to empathy development (Lin et al., 2023). In addition, these findings provide further insight into the Stereotype Content Model by suggesting that adolescents’ evaluations of older adults on the dimensions of warmth and competence may not solely reflect broader societal narratives, but may also be associated with differences in socio-emotional development. In the Chinese cultural context, judgments of warmth may be particularly sensitive to emotional experiences within the family, whereas competence evaluations may additionally reflect culturally embedded expectations regarding older adults’ social roles and contributions.

Finally, the present findings further suggest that empathy may be more strongly associated with positive stereotypes of older adults when adolescents have more frequent opportunities for meaningful intergenerational interaction. Under such conditions, older adults are more likely to be perceived as unique individuals rather than abstract social categories, allowing empathic tendencies to be expressed in actual interpersonal encounters. By contrast, when intergenerational contact is limited, empathy may have fewer opportunities to translate into more positive perceptions of older adults. Rather than suggesting that empathy operates independently, these findings indicate that its association with age stereotypes may depend on the availability of supportive interaction contexts, thereby extending previous research on the joint roles of empathy and intergroup contact.

The present findings should also be interpreted within the broader Chinese cultural context. Confucian traditions emphasize filial piety and respect for older adults as important social norms. Nevertheless, the persistence of negative age stereotypes among some adolescents suggests that cultural expectations alone may not be sufficient to foster positive perceptions of older adults. Instead, broader cultural values may be internalized through everyday family interactions. In emotionally supportive families, filial values may be reinforced through empathic communication and positive parent–child relationships, whereas emotionally unsupportive parenting may provide fewer opportunities for adolescents to internalize these values. Therefore, the present findings suggest that macro-level cultural norms and micro-level family experiences may jointly contribute to adolescents’ perceptions of older adults.

### 5.1 Theoretical contributions

First, this study extends age stereotype research by highlighting family experiences as one important developmental context associated with adolescents’ perceptions of older adults. While previous studies have primarily emphasized macro-level influences such as media representations, social norms, and intergenerational contact, our longitudinal findings suggest that family socialization may also contribute to individual differences in age stereotypes. Rather than positioning family as an exclusive or foundational mechanism, the present study suggests that family experiences should be considered alongside other important socialization contexts, including schools, peers, and the media. By applying the warmth and competence dimensions of the Stereotype Content Model, this study provides a more nuanced examination of adolescents’ evaluations of older adults.

Second, this study extends current understanding of the relationship between empathy and age stereotypes by considering the developmental role of family experiences. Whereas previous research has often conceptualized empathy as a relatively stable individual characteristic or an immediate predictor of prejudice reduction, our findings suggest that adolescents’ empathic capacities may be associated with parenting experiences characterized by emotional responsiveness or emotional neglect. This developmental perspective complements existing research by suggesting that socio-emotional experiences within the family may influence how adolescents perceive members of other social groups.

Third, this study provides a theoretical extension to research on intergroup contact by examining intergenerational contact as a boundary condition for the association between empathy and age stereotypes. Previous studies have generally examined empathy and intergroup contact as separate predictors of prejudice reduction or have considered contact as an antecedent of empathy. Our findings suggest that empathy may be more strongly associated with positive perceptions of older adults when adolescents have greater opportunities for meaningful intergenerational interaction. Rather than revising existing theory, this finding extends current perspectives by highlighting the potential interaction between individual socio-emotional capacities and social interaction contexts.

Finally, this study contributes to culturally informed family research by examining these relationships within the Chinese cultural context. Although Confucian values emphasize filial piety and respect for older adults, our findings suggest that broader cultural norms may interact with everyday family experiences in shaping adolescents’ perceptions of older adults. This highlights the importance of considering both macro-level cultural values and micro-level family processes when examining the development of age stereotypes.

### 5.2 Practical implications

The present findings offer several practical implications, although the effectiveness of the proposed strategies should be examined in future intervention-based research.

For parenting educators and family practitioners, the findings suggest that it may be valuable to emphasize emotional responsiveness in parenting interventions, particularly for families at risk of engaging in negative parenting practices. Parenting programs could consider incorporating training in emotional validation, active listening, and empathic communication to facilitate adolescents’ socio-emotional development. Given that a substantial proportion of participants in the present study were from rural areas, community-based parenting support delivered through schools or local family service organizations may provide accessible opportunities for caregiver education.

For educators and school-based mental health professionals, the findings suggest that empathy training may be more effective when combined with opportunities for meaningful intergenerational interaction. Schools could consider integrating structured intergenerational activities, such as service-learning projects, digital literacy programs involving older adults, or oral history initiatives, to provide adolescents with opportunities to apply empathic skills in authentic social contexts. However, whether these approaches effectively reduce age stereotypes requires further empirical evaluation.

For community organizations and policymakers, the findings suggest that creating environments that facilitate positive intergenerational interaction may contribute to improving adolescents’ perceptions of older adults. Community centers, volunteer programs, and shared recreational activities could provide opportunities for sustained intergenerational engagement. Particular attention may also be given to adolescents who experience limited emotional support within the family or have fewer opportunities for intergenerational interaction. Nevertheless, the effectiveness and generalizability of these proposed interventions should be verified through future experimental and intervention-based studies before firm policy recommendations can be made.

### 5.3 Limitation

While this study makes important contributions, several limitations point to promising directions for future research. First, our measure of intergenerational contact focused solely on cohabitation duration with grandparents. Future research could develop more nuanced measures that capture the quality, frequency, and diversity of intergenerational interactions, as well as contact with non-familial older adults. More importantly, this variable was only measured at time point 1, which weakened its dynamic interpretation. Second, although our findings demonstrate that negative parenting is associated with adolescents’ stereotypes of older adults, we did not assess adolescents’ evaluations of other social groups. Therefore, we cannot determine whether this association reflects age-specific stereotyping or a broader tendency toward negative social evaluation. Third, our sample is limited to a specific school district in a single province of China. Moreover, the majority of the samples come from rural areas, which to some extent limits the generalizability of the interpretation. Future research should replicate these findings in other cultural contexts to determine the generalizability of our model, as well as examine how cultural values such as filial piety moderate the relationships we observed. Fourth, when analyzing the common method bias, only the single-factor method was adopted. In the future, more methods can be used, such as latent factor analysis, to enhance its robustness. Finally, the control variables have limitations. For instance, the educational level of parents and previous experiences in caring for the elderly have not been measured and incorporated into the model.

## 6 Conclusion

In conclusion, this three-wave longitudinal study of 2,011 Chinese adolescents suggests that negative parenting practices are associated with less positive stereotypes of older adults and that empathy may help explain this association. The findings also indicate that the positive association between empathy and age stereotypes is stronger among adolescents reporting higher levels of intergenerational contact, suggesting that meaningful contact may provide a supportive context in which empathic tendencies are more readily reflected in perceptions of older adults.

This study extends current understanding by applying Social Learning Theory to examine how family experiences and social interactions are associated with adolescents’ perceptions of older adults. Rather than suggesting that family experiences are the sole influence, the findings highlight family as one important developmental context alongside schools, peers, and broader social environments. As the sample was drawn from adolescents in Henan, China, the findings should be interpreted with appropriate caution, and future research using experimental and cross-cultural designs is needed to further examine the generalizability of these associations.

## Data Availability

The raw data supporting the conclusions of this article will be made available by the authors, without undue reservation.
